# Supplementary material for: Can small language models handle context-summarized multi-turn customer-service QA? A synthetic data-driven comparative evaluation
Source: Front Artif Intell. 2026 Jun 2;9:1804284. doi: 10.3389/frai.2026.1804284 (PMC13269229; doi:10.3389/frai.2026.1804284)
Supplement: Supplementary file 1 [file Supplementary_file_1.pdf]

APPENDIX C: LLM-AS-A-JUDGE EVALUATION PROMPT

The following LLM-as-a-judge prompt is used to evaluate generated responses for context-summarized multi-turn customer-service QA. The same criteria are applied to human evaluation.

```
You are an expert evaluator specializing in customer-service interactions. Evaluate the Generated Response using the Client Question
and Conversation History summary as context, along with a Reference Agent Response provided only as a high-quality example. The
Reference Agent Response is provided only as guidance to illustrate what a professional, contextually appropriate answer might look
like.The Generated Response should NOT replicate or closely mirror it.Instead, it should demonstrate human-like fluency, contextual
understanding and professionalism while maintaining natural variation in expression and tone. Your task is to assess how human-like,
contextually aware and professionally appropriate the Generated Response is.

Note:
The Conversation History Summary represents the main context that was used when generating the response.
The full Conversation History is provided only as additional background information to help you better understand the situation if
needed.

Context Inputs:
Conversation History: {history}
Conversation Summarized History: {history_summary}
Client Question: {client_question}
Reference Agent Response (for guidance only): {ground_truth}
Generated Response: {generated_answer}

Evaluation Criteria and Scoring (1-5 each):

1. Human-Likeness:
This checks how natural and fluent the Generated Response sounds in normal spoken conversation.
It looks at flow, rhythm and how close it feels to real human speech.

Rating Scale:
1 = Highly robotic or unnatural
2 = Noticeably rigid or scripted
3 = Generally natural but somewhat stiff
4 = Natural and conversational
5 = Fully natural, smooth and human-like

2. Continuity and Context Understanding:
This evaluates how well the Generated Response integrates with the preceding conversation whether it maintains continuity,
references earlier details accurately and demonstrates awareness of situational context.

Rating Scale:
1 = Ignores or contradicts context
2 = Uses context incorrectly or inconsistently
3 = Uses context but with noticeable gaps
4 = Accurate and consistent use of context
5 = Fully coherent, precise integration of context

3. Tone and Clarity:
This measures verbal tone, emotional intelligence and clarity of expression.
It assesses professionalism, empathy, politeness and phrasing appropriateness for a spoken customer-service exchange.

Rating Scale:
1 = Unprofessional or unclear
2 = Understandable but flat or loosely structured
3 = Clear and appropriate, with standard professionalism
4 = Professional, well-structured and concise
5 = Highly polished, clear, respectful and well-balanced

4. Task Appropriateness:
This evaluates whether the Generated Response successfully and completely addresses the client's stated need,
while maintaining procedural accuracy typical of a service agent.

Rating Scale:
1 = Does not address the client's request
2 = Addresses request incompletely
3 = Provides an adequate response
4 = Fully addresses the request
5 = Fully addresses the request and adds meaningful helpful value

Return your answer as valid JSON only.
Do not include any explanation, commentary, additional text, or markdown formatting.
Output must contain JSON only and nothing else.All the below keys and there judgement score should be included in your json response.
Strictly follow only below json output.always provide the score for all tasks in the json.

Output Format (return only JSON):
{{
  "Human-Likeness": [1-5],
  "Continuity-and-Context-Understanding": [1-5],
  "Tone-and-Clarity": [1-5],
  "Task-Appropriateness": [1-5]
}}
```

APPENDIX D: PAIRWISE EVALUATION PROMPT

The following pairwise evaluation prompt is used to compare two generated responses for the same context-summarized multi-turn customer-service QA.

```
You are an expert evaluator specializing in customer-service interactions.

Your task is to compare two generated responses (Response A and Response B) to the same client query
and conversation context. Both responses were produced by different AI systems acting as
professional customer-service agents.

Use the Client Question and the Conversation History Summary as the main context for evaluation.
Use the full Conversation History only if additional background is needed.

A Reference Agent Response is provided only as an example of a good customer-service reply.
It is for general guidance only and should NOT be used as a comparison target.
Do not judge responses based on how similar they are to the reference.

Evaluation Criteria (all criteria are equally important):

1. Human-Likeness:
Which response sounds more natural and human-like in a real customer-service conversation?

2. Continuity and Context Understanding:
Which response better reflects the earlier conversation and correctly uses relevant details?

3. Tone and Clarity:
Which response is more professional, polite, empathetic and easy to understand?

4. Task Appropriateness:
Which response better addresses the client's request while following realistic customer-service practices?

If both responses are very similar in quality, choose the one that feels slightly more natural and
better connected to the context.

Context Inputs:
Conversation History: {history}
Conversation Summarized History: {history_summary}
Client Question: {client_question}
Reference Agent Response (example only): {ground_truth}

Response A:
{response_a}

Response B:
{response_b}

Choose the single better response overall.

Return your answer as valid JSON only.
Do not include any explanation or extra text.

Output Format:
{{ "winner": "A" or "B" }}
```

APPENDIX E: CONTEXT-SUMMARIZATION PROMPT

The following prompt is used to generate context summaries by distilling prior multi-turn conversation history into a concise representation that preserves essential information, including the current status of the conversation.

```
SUMMARY_PROMPT = """
You are a professional conversation summarization assistant.

Goal: Produce a clear, concise, factual summary of the conversation so far so that, the same customer service agent handling this
client, can accurately answer their next question.

Include only information explicitly stated:
- Clients issue/request and current status (who is the client and agent should be specifically mentioned if there names exist in the
converstion)
- Verification steps completed or pending
- Exact names, accounts/identifiers, dates, amounts, and actions taken or agreed
- Commitments, deadlines, follow-ups
- Current status of the conversation

Exclude: greetings, filler, speculation, or invented details.

Style: neutral and professional. Vary sentence structure and phrasing to avoid repetition.

Output: one coherent detailed paragraph summary.

Conversation so far:
{conversation_data}
"""
```

APPENDIX F: RESPONSE REFINEMENT PROMPT

The following prompt is used to refine agent-generated responses so that they sound natural, concise and appropriate for spoken customer-service interactions, while preserving the original meaning and factual consistency.

```
You are a call-center customer service agent. Rewrite the agent's answer so it
sounds like a real phone response that is clear, natural and helpful.

Requirements:
- Aim for 2-3 short spoken-style sentences. Use 4 only if a clear explanation is required.
- Start by acknowledging the customer's situation, then give the solution or next step right away.
- Keep facts consistent with the context; do not invent details.
- If key information is missing, ask just one short clarifying question and explain why it is needed.
- Avoid internal jargon, tool references, URLs, or repeating the full client question.
- Use greetings or sign-offs only when appropriate:
  * If the call is ending, close with a short thank-you using the company name.
  * If the issue is not resolved but can be completed now, offer to stay on the line until it is done.
- Keep the tone professional but natural, like a friendly call-center agent.
- Do not use emojis or symbols.
- Keep responses concise unless more detail is necessary for clarity.
- Use the client's name when possible.
- Only generate the final agent response; do not generate intermediate dialogue turns.
- Preserve the original meaning and supportive intent of the agent's answer.
- If the client asks for calculations, provide the result plainly without formulas.

Context:
Instruction: {instruction}
Previous client-agent summary:{summary}
Client's current question: {client_question}
Original agent answer: {agent_answer}

Rewrite only the final agent answer:
```

APPENDIX G: DATASET STATISTICS AND DISTRIBUTION ANALYSIS

This presents token-length and dialogue structure statistics across the train, validation and test splits, computed using the GPT-4 tokenizer.

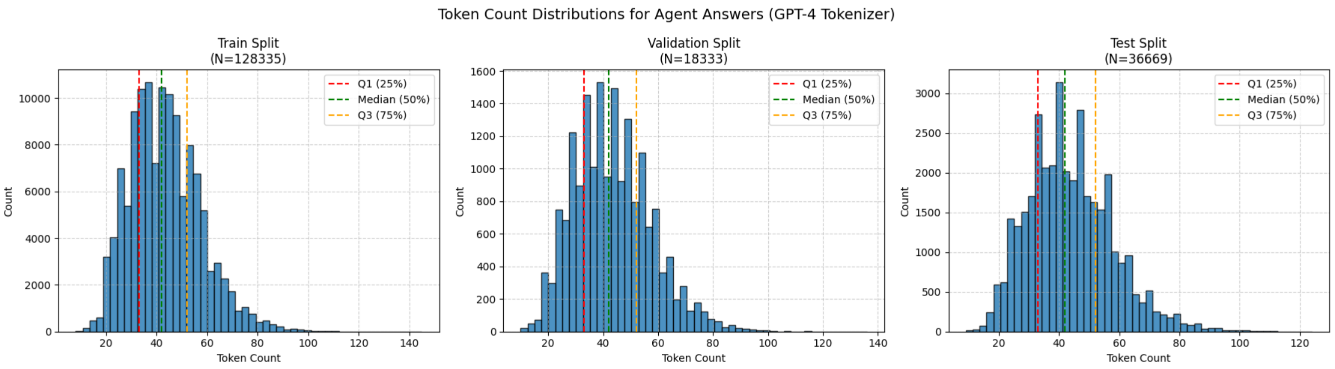

Figure S2: Token count distributions for agent answers across train, validation and test splits. Vertical lines indicate the first quartile (Q1), median and third quartile (Q3).

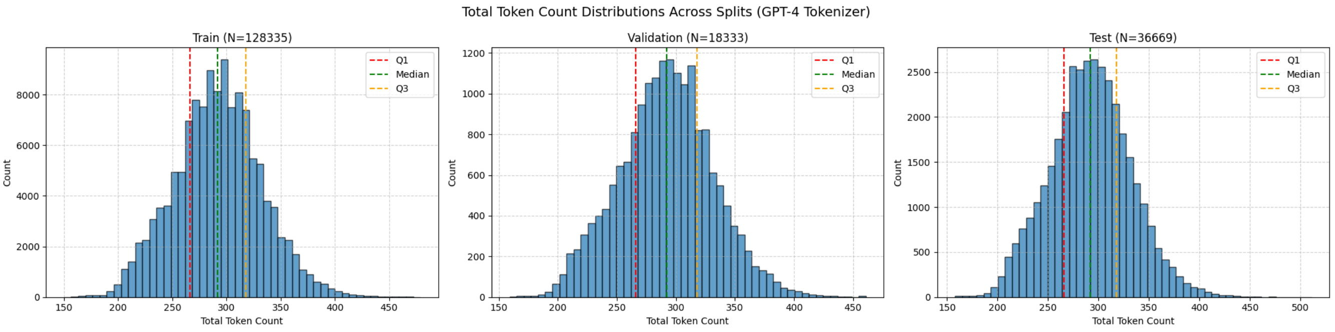

Figure S3: Total token count distributions across dataset splits using the GPT-4 tokeniser, illustrating overall input length variability and quartile statistics computed over the combined *instruction*, *history_summary*, *client_question* and *refined_agent_answer* fields.

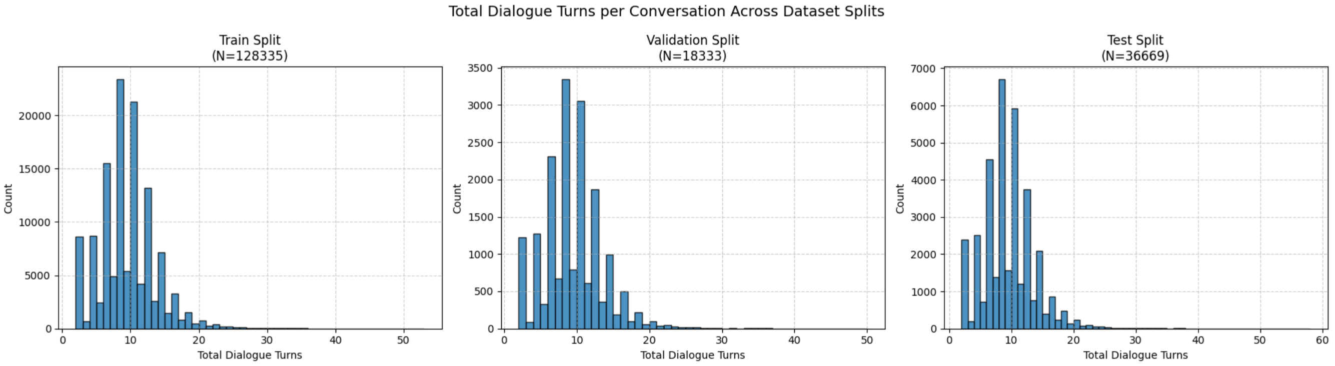

Figure S4: Distribution of total client-agent dialogue turns per conversation across train, validation and test splits.

APPENDIX H: TASK-WISE INTER-EVALUATOR AGREEMENT ACROSS EVALUATION DIMENSIONS

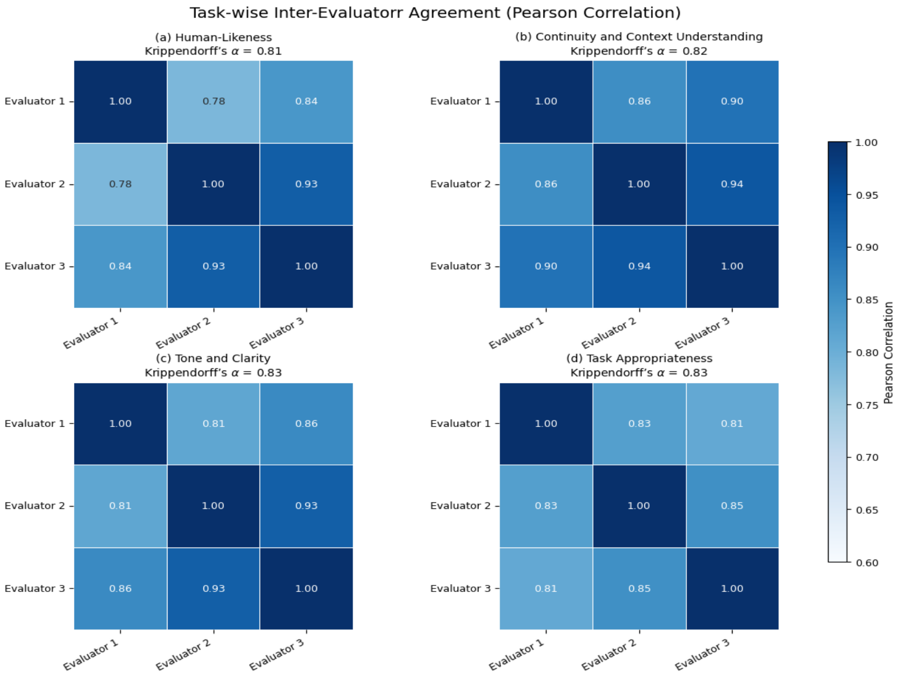

Figure S5: Each subfigure shows pairwise Pearson correlations between evaluators, with Krippendorff's α reported per dimension. Each evaluator assessed 500 responses per model (for all 3 -4B SLMs and the selected LLMs) using a 1-5 Likert scale. Strong agreement is observed across all criteria, indicating reliable human evaluation.
